# Two cases of treatment-resistant erythema nodosum responding favorably to infliximab in the absence of gastrointestinal disease

**DOI:** 10.1016/j.jdcr.2025.09.008

**Published:** 2025-09-19

**Authors:** Nicole Nowak, Maria L. Mihailescu, Kyle T. Amber

**Affiliations:** a Rush Medical College, Chicago, Illinois; b Department of Dermatology, Rush University Medical Center, Chicago, Illinois

**Keywords:** erythema nodosum, inflammatory bowel disease, infliximab, TNF-α, tumor necrosis factor

## Introduction

Erythema nodosum (EN) is an immune-mediated septal panniculitis characterized by tender, erythematous nodules, typically of the lower legs, associated with infectious, inflammatory, and malignant pathologies. However, most cases of EN are idiopathic.^1^ Chronic EN can be particularly challenging to manage, and treatment options for recalcitrant disease are limited. Data from the gastroenterology literature supports the utility of tumor necrosis factor (TNF)-α inhibitors for EN in the setting of underlying inflammatory bowel disease (IBD).^2^ However, reports of TNF-α inhibition in EN independent of IBD are sparse.^3^ Herein, we present 2 cases of treatment-resistant EN, not associated with IBD, responding favorably to infliximab.

## Case 1

**Fig 1:**
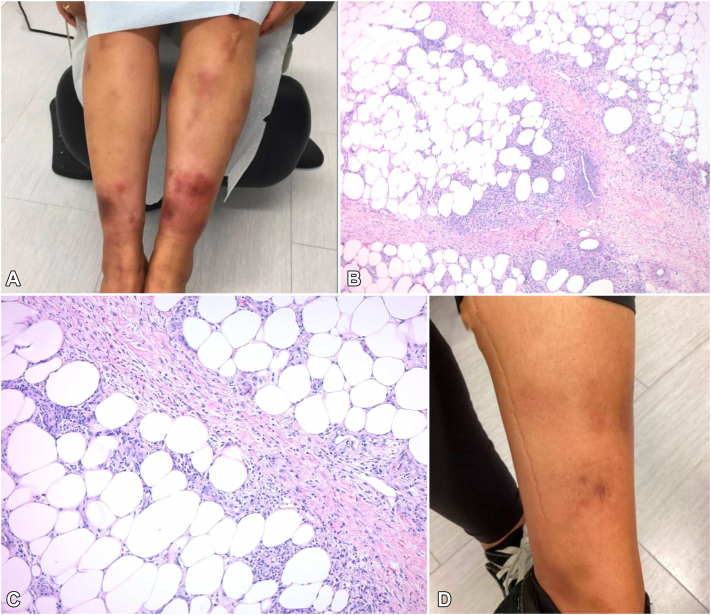
**A,** Violaceous and erythematous nodules and plaques on the lower legs in patient 1 prior to infliximab; **(B)** Septal panniculitis with fibrosis and granulomatous inflammation (40×); **(C)** Lymphocyte and histocyte-rich inflammation with septal fibrosis; **(D)** Resolution of tender nodules with postinflammatory hyperpigmentation 6-months following infliximab loading.

The first case describes a 38-year-old female with no history of IBD who presented with a 2-year history of biopsy-proven recurrent EN that developed following a COVID-19 infection (Fig 1, *A-C*). Clinical examination revealed scattered indurated dusky erythematous plaques with areas of faint desquamation on both legs. A punch biopsy demonstrated septal panniculitis with fibrosis and granulomatous inflammation. Laboratory tests, including rheumatoid factor, antistreptolysin antibody, and QuantiFERON, were all within normal limits. A comprehensive autoimmune panel and chest X-ray were also performed to evaluate for underlying inflammatory conditions, including sarcoidosis and connective tissue disease, which were all unremarkable. Over 2 years, the patient failed multiple systemic therapies including colchicine (0.6 mg twice daily), dapsone (100 mg), pentoxifylline (400 mg twice daily), super saturated potassium iodide (300 mg thrice daily), and multiple nonsteroidal anti-inflammatories medications in addition to high-potency topical corticosteroids. While she was responsive to oral prednisone (up to 60 mg daily), the disease rapidly flared upon taper over 3-4 weeks. Her pain management required intermittent use of opioids. A pregnancy test was obtained and negative. The patient was subsequently started on infliximab at a dose of 7.5 mg/kg every 6 weeks (loading doses at weeks 0, 2, and 6). By week 6, she experienced significant pain relief and improvement in nodules, erythema, and induration. At the 4-month follow-up, she reported near resolution of nodules and discomfort (Fig 1, *D*), with mild breakthrough lesions occurring 5 weeks following each infusion. She opted against increased infusion frequency and has remained well-controlled at 6-month follow-up.

## Case 2

**Fig 2:**
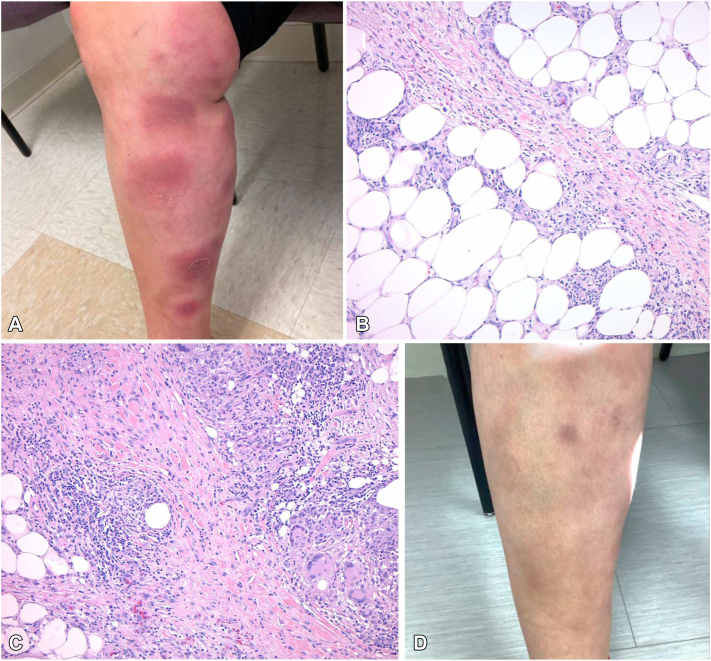
**A,** Erythematous, tender nodules on the bilateral anterior legs in patient 2 prior to infliximab; **(B)** Septal panniculitis with granulomatous inflammation and fibrosis (40×); **(C)** Marked fibrosis and widening of the septae by histiocyte-rich inflammation composed of numerous large granulomas with multinucleated giant cells and epithelioid histiocytes (100×); **(D)** Resolution of tender nodules with postinflammatory hyperpigmentation 4-months following infliximab loading.

The second case describes a 45-year-old female with no history of IBD who presented with a 16-year history of biopsy-proven EN (Fig 2, *A*-*C*). She experienced frequent and recurrent painful indurated plaques primarily limited to the lower legs. An incisional biopsy revealed marked fibrosis and significant widening of the septae within the subcutis, characterized by histiocyte-rich inflammation. The inflammatory process included numerous large granulomas composed of epithelioid histiocytes and multinucleated giant cells, some of which encircled necrotic adipocytes. Additionally, there was a prominent lymphocytic infiltrate with extension of the inflammation into the surrounding fat lobules. Her medical history was otherwise notable for multiple miscarriages, but a comprehensive workup for connective tissue disease was negative, including normal levels of anticardiolipin, beta-2 glycoprotein 1, lupus anticoagulant, and complement levels (C3 and C4). A chest X-ray showed no evidence of sarcoidosis or pulmonary involvement. A QuantiFERON-TB Gold was also negative. The patient’s EN was recalcitrant to nonsteroidal anti-inflammatories, salicylates, super saturated potassium iodide (300 mg thrice daily), colchicine (1.2 mg twice daily), dapsone (100 mg daily), hydroxychloroquine (200 mg twice daily), methotrexate, as well as topical and intralesional corticosteroids. The patient responded to prednisone (up to 60 mg) but would rapidly flare upon tapering below 15 mg. Her pain management also required opioids. The patient was started on infliximab at 5 mg/kg for 3 loading doses (0, 2, and 6 weeks), followed by infusions every 8 weeks thereafter. Following loading dose, the patient reported resolution of pain, no new lesions, and improvement in old lesions. At the 6-month follow-up, the patient reported significant durable improvement with infrequent and mild flares (Fig 2, *D*).

## Discussion

While several reports have demonstrated the efficacy of anti-TNF-α agents in IBD-associated EN, few cases of idiopathic EN have been described, and none to our knowledge, with infliximab.^3^^,^^4^ We initially pursued adalimumab for both patients, given its stronger evidence base in idiopathic EN and the convenience of subcutaneous administration.^3^ However, insurance coverage was denied. As such infliximab was pursued as a second-line TNF-α inhibitor and approved. A dose of 7.5 mg/kg, with loading doses at weeks 0, 2, and 6, followed by maintenance infusions every 6 weeks was requested in both cases, based on dosing regimens previously reported in the literature for other inflammatory conditions including hidradenitis suppurativa and pyoderma gangrenosum.^5^^,^^6^ In Case 2, the patient received approval only for 5 mg/kg with every 8 weeks maintenance dose. We present these cases to highlight its potential as an effective therapeutic option in recalcitrant, non–IBD-associated EN.

The mechanism of EN is complex, thought to represent a hypersensitivity response to various antigens. Pathogenesis may involve immune complex deposition in the septal venules of subcutaneous fat, triggering TNF-α production, neutrophil recruitment, and reactive oxygen species formation.^7^ Granuloma formation is also frequently detected histologically. A prospective study characterizing the cytokine network in patients with EN revealed an overexpression of TNF-α in the serum.^8^ Additionally, the TNF-α II allele polymorphism was shown to be significantly associated with the development of EN in the setting of sarcoidosis.^9^

## Conclusion

These favorable outcomes suggest that TNF-α inhibition may offer a valuable therapeutic option for managing EN outside of the setting of IBD, particularly in treatment-resistant cases. While paradoxical induction of EN has been reported, these instances have occurred exclusively in the setting of other systemic inflammatory conditions such as IBD, ankylosing spondylitis, and rheumatoid arthritis. Although further prospective studies are warranted, our findings support intravenous infliximab as a promising therapy for recalcitrant idiopathic EN.

## Conflicts of interest

None disclosed.
